# An Off-Grid Turbo Channel Estimation Algorithm for Millimeter Wave Communications

**DOI:** 10.3390/s16101562

**Published:** 2016-09-22

**Authors:** Lingyi Han, Yuexing Peng, Peng Wang, Yonghui Li

**Affiliations:** 1 Wireless Signal Processing and Networks (WSPN) Lab, Key Lab of Universal Wireless Communications, Ministry of Education, Beijing University of Posts and Telecommunications, No. 10 Xitucheng Road, Beijing 100876, China; hanlingyi@bupt.edu.cn; 2 Huawei Technologies, Sweden AB, Kista 164 40, Sweden; wp_ady@hotmail.com; 3 Center of Excellence in Telecommunication, University of Sydney, Sydney NSW 2006, Australia; yonghui.li@sydney.edu.au

**Keywords:** channel estimation, millimeter wave communication, off-grid algorithm

## Abstract

The bandwidth shortage has motivated the exploration of the millimeter wave (mmWave) frequency spectrum for future communication networks. To compensate for the severe propagation attenuation in the mmWave band, massive antenna arrays can be adopted at both the transmitter and receiver to provide large array gains via directional beamforming. To achieve such array gains, channel estimation (CE) with high resolution and low latency is of great importance for mmWave communications. However, classic super-resolution subspace CE methods such as multiple signal classification (MUSIC) and estimation of signal parameters via rotation invariant technique (ESPRIT) cannot be applied here due to RF chain constraints. In this paper, an enhanced CE algorithm is developed for the off-grid problem when quantizing the angles of mmWave channel in the spatial domain where off-grid problem refers to the scenario that angles do not lie on the quantization grids with high probability, and it results in power leakage and severe reduction of the CE performance. A new model is first proposed to formulate the off-grid problem. The new model divides the continuously-distributed angle into a quantized discrete grid part, referred to as the integral grid angle, and an offset part, termed fractional off-grid angle. Accordingly, an iterative off-grid turbo CE (IOTCE) algorithm is proposed to renew and upgrade the CE between the integral grid part and the fractional off-grid part under the Turbo principle. By fully exploiting the sparse structure of mmWave channels, the integral grid part is estimated by a soft-decoding based compressed sensing (CS) method called improved turbo compressed channel sensing (ITCCS). It iteratively updates the soft information between the linear minimum mean square error (LMMSE) estimator and the sparsity combiner. Monte Carlo simulations are presented to evaluate the performance of the proposed method, and the results show that it enhances the angle detection resolution greatly.

## 1. Introduction

Thanks to the large bandwidth available at millimeter wave (mmWave) frequencies, mmWave communication technology has become a promising technology to meet the experientially increasing demands of future wireless networks [1]. The small wavelength at the mmWave band enables the integration of a massive number of antennas at both the transmitter and receiver. The directional beamforming technique can be used to achieve a sufficient link margin to compensate for the severe propagation attenuation in mmWave bands [2,3]. Since the channel state information, especially the angle of departure (AoD) and angle of arrival (AoA), are essential for the beamforming and coherent detection, CE with high resolution and efficiency is one of the key requirements in mmWave systems. Due to the high cost and high power consumption of mmWave radio frequency (RF) devices, only a very limited number of RF chains can be integrated in the mmWave systems, which results in much less RF chains than antennas. Therefore, classic angle estimation methods with super-resolution, such as multiple signal classification (MUSIC) and estimation of signal parameters via rotation invariant technique (ESPRIT)-based algorithms [4,5], cannot be directly applied in mmWave systems because these methods require the channel observations at each receive antenna in the digital domain. However, in the mmWave systems, the received signals at each antenna are firstly combined in the analog domain and thus no direct output information from each antenna is available for these classic angle estimation methods. Therefore, new CE algorithms with high-resolution should be developed and tailored to mmWave communications.

Recently, many research efforts have been devoted to the CE for the mmWave systems [6,7,8,9,10]. As adopted in IEEE 802.15.3c standard [6], a polling mechanism is employed to select the best beam vector pair from the known codebooks with *p* beams at the transmitter and *q* beams at the receiver. This method consumes pq time slots to achieve angle resolution O(1/p) and O(1/q). In [7], an adaptive compressive sensing (ACS) method has been developed, which iteratively bisections the beam space and tries to find the grid that the path falls in via space match filtering. It needs $2L^{3}log_{2}\left(p/L\right)+2L^{3}log_{2}\left(q/L\right)$ time slots to achieve the resolution O(1/p) at the transmitter and O(1/q) at the receiver for an mmWave channel with *L* paths. This large time slot payload is unacceptable in practical mobile communication systems with massive antenna arrays, especially in the outdoor wireless coverage scenarios. So far, most existing mmWave CE methods, such as [8,9,10], are based on the on-grid channel model, i.e., all path angles are assumed to be located at the quantization grids exactly, which is not the case in practical systems because the angles are usually continuously distributed. When path angles do not lie on the quantization grids, which we refer to as the “off-grid” channel model, a power leakage effect happens and results in the degradation of CE performance [11]. Since the product of the number of simultaneously supported multi-users and the number of the multi-streams depends on the RF chains, it is preferable that higher angle resolution is achieved by advanced channel estimation methods to separate densely distributed users such that spatial multiplexing methods can be employed to support more users [12]. In this ”off-grid” case, the gap to the theoretic lower bound is pretty large, and more antennas are required to enhance the resolution of angle estimation at the cost of more hardware and antennas [13]. Therefore, a new method is necessary to enhance the angle detection performance. So far, few works concentrate on the off-grid problem in the mmWave systems with RF constraints. In [14], the continuous angle estimation was formulated as a sparse regularization problem by taking into account the noises in both the observations and the dictionary by modeling the grid points as noisy, which is hard to be applied in CE because the spectral norm of the noise is much bigger than that of the dictionary. In [15], a continuous basis-based compressed sensing (CS) method was proposed to solve the reconstruction problem in sensing applications where the atoms of the dictionary are used to describe a continuous field, such as frequency and angle. However, the first order approximation of Taylor expansion for the array steering vector in the multi-antenna systems is hard to formulate.

In this paper, we study the off-grid problem and propose an enhanced method to improve the resolution of angle estimation in mmWave systems with massive antenna arrays and RF chain constraints. Firstly, we formulate the signal model to describe the off-grid problem in the angle quantization of the mmWave channel, which decomposes each continuously-distributed angle into the integral grid part and fractional off-grid part. Accordingly, an iterative off-grid turbo CE (IOTCE) algorithm is developed to renew and upgrade the CE between the integral grid and fractional off-grid angle information under the Turbo principle. By fully exploiting the sparse structure of mmWave channels, a soft-decoding based CS method, termed improved turbo compressed channel sensing (ITCCS), is developed to estimate the integral grid angle information, which iteratively updates the soft information between the linear minimum mean square error (LMMSE) estimator and the sparsity combiner. Simulation results show that the proposed CE method can achieve a higher resolution than the existing ones.

The rest of the paper is organized as follows. The mmWave system model is introduced in Section 2. Then, a new off-grid formulation is specified in Section 3 and the proposed IOTCE algorithm is detailed in Section 4. Section 5 presents the simulation results and Section 6 concludes the paper.

*Notations*: Fonts A, a, *a* and A denote a matrix, a column vector, a scalar and a set, respectively. $A^{T}$ and $A^{H}$ are the transpose and Hermitian of A. $1_{N}\in C^{N\times 1}$ is an all-ones vector and I is an identity matrix. $e_{N}\left(i\right)\in C^{N\times 1}$ is a unit vector with 1 at its *i*-th entry and 0 elsewhere. The operator ⊗ are used to denote the Kronecker product. Vect(A) is the matrix vectorization operator that transforms a matrix into a vector by stacking its column vectors, and Diag(a) stands for a diagonal matrix with the entries of a on its diagonal. ${\left[A\right]}_{R,P}$ consists of row vectors and column vectors of the matrix A whose indices contained in the set R and P, respectively. E(·) represents the expectation. δ(·) is the Dirac delta function and I(·) is the indicator function.

## 2. System Model

**Figure 1:**
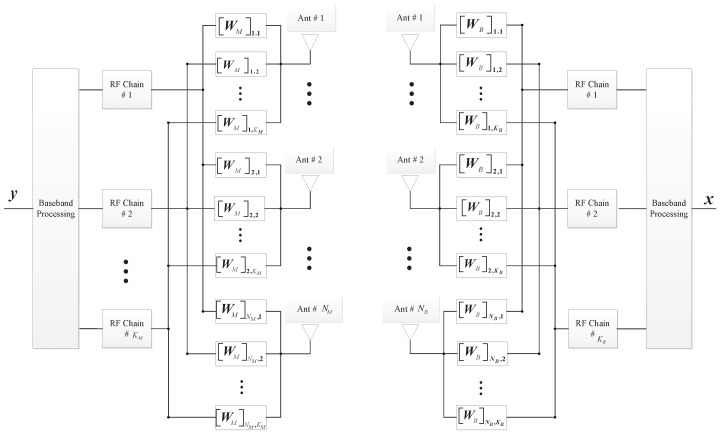
Block diagram of structure with beamforming at the base station (BS) and combining at the mobile station (MS).

According to [16], typical mmWave channels feature much stronger line-of-sight (LoS) path than non-LoS (NLoS) paths, and the numerical results in [17] show that approximating the sparse mmWave channel with only one LoS path results in a little capacity loss when employing the steerable directional antennas. Thus, single-path mmWave channel model is used in this paper. When the mmWave channel cannot be approximated as a single-path channel, the proposed algorithm cannot be applied directly. New algorithms need to be developed to deal with independently but not identically distributed multi-paths. Consider a downlink mmWave system consisting of a base station (BS) and a mobile station (MS), where the BS is configured with $N_{B}$ antennas and $K_{B}$ RF chains with $K_{B}\ll N_{B}$ while the MS is configured with $N_{M}$ antennas and $K_{M}$ RF chains with $K_{M}\ll N_{M}$, as shown in Figure 1. The BS applies the beamforming matrix $W_{B}\in C^{N_{B}\times K_{B}}$ and the MS employs the matrix $W_{M}\in C^{N_{M}\times K_{M}}$ to combine the received signal $y\in C^{K_{M}\times 1}$. The received signal at the MS can be written as:

(1) $$\begin{array}{c} y=W_{M}^{T}HW_{B}x+z \end{array}$$

where $H\in C^{N_{M}\times N_{B}}$ is the channel matrix, $x\in C^{K_{B}\times 1}$ represents the transmitted pilot signal, and $z\in C^{K_{N}\times 1}$ is the Gaussian noise with $E\left({\mathrm{zz}}^{H}\right)=\sigma^{2}I$.

In this paper, we also assume:

- uniform linear arrays (ULAs) with half-wavelength spacing are deployed at both the BS and MS;
- far-field scattering and block-fading are held, which means the signal waves arrive at different antennas with the same fading amplitudes but distinct phases and the channel fadings are kept constant during the CE precedure.

Under these assumptions, the single-path channel between the BS and MS can be expressed as:

(2) $$\begin{array}{c} H=\beta a_{M}\left(\theta_{M}\right)a_{B}^{T}\left(\theta_{B}\right) \end{array}$$

where *β* is the fading weight, $\theta_{B}\in \left[-\frac{\pi }{2},\frac{\pi }{2}\right)$, and $\theta_{M}\in \left[-\frac{\pi }{2},\frac{\pi }{2}\right)$ are, respectively, AoD and AoA. $a_{B}$ and $a_{M}$ are the array steering vector at the BS and the combining vector at the MS, respectively. Denoting $\frac{\sin\theta_{B}}{2}$ by $\phi_{B}\in \left[-\frac{1}{2},\frac{1}{2}\right)$ and $\frac{\sin\theta_{M}}{2}$ by $\phi_{M}\in \left[-\frac{1}{2},\frac{1}{2}\right)$, $a_{B}$ and $a_{M}$ can be written as:

(3) $$\begin{array}{c} a_{B}\left(\phi_{B}\right)=\frac{1}{\sqrt{N_{B}}}{\left[1\;e^{j2\pi \phi_{B}}\cdots e^{j2\pi \left(N_{B}-1\right)\phi_{B}}\right]}^{T} \end{array}$$

(4) $$\begin{array}{c} a_{M}\left(\phi_{M}\right)=\frac{1}{\sqrt{N_{M}}}{\left[1\;e^{j2\pi \phi_{M}}\cdots e^{j2\pi \left(N_{M}-1\right)\phi_{M}}\right]}^{T} \end{array}$$

The (m,n)-th element of the channel can be rewritten as:

(5) $$\begin{array}{cc} {\left[H\right]}_{m,n} & =\beta e^{j2\pi \left(m-1\right)\phi_{M}}e^{j2\pi \left(n-1\right)\phi_{B}}\\ & =\int_{-\frac{1}{2}}^{\frac{1}{2}}\int_{-\frac{1}{2}}^{\frac{1}{2}}h\left({\tilde{\phi }}_{M},{\tilde{\phi }}_{B}\right)e^{j2\pi \left(m-1\right){\tilde{\phi }}_{M}}e^{j2\pi \left(n-1\right){\tilde{\phi }}_{B}}\;d{\tilde{\phi }}_{M}\;d{\tilde{\phi }}_{B} \end{array}$$

where $h\left({\tilde{\phi }}_{M},{\tilde{\phi }}_{B}\right)=\beta \delta \left({\tilde{\phi }}_{M}-\phi_{M}\right)\delta \left({\tilde{\phi }}_{B}-\phi_{B}\right)$ is the channel impulse response (CIR). By discretizing Equation (5) at the angle period $\left[-\frac{1}{2},\frac{1}{2}\right)$,

(6) $$\begin{array}{c} {\left[H\right]}_{m,n}\left(k,i\right)=\sum_{k=-N_{M}/2}^{N_{M}/2-1}\sum_{i=-N_{B}/2}^{N_{B}/2-1}h\left(k/N_{M},i/N_{B}\right)e^{j2\pi \left(m-1\right)\left(k+\delta_{M}\right)/N_{M}}e^{j2\pi \left(n-1\right)\left(i+\delta_{B}\right)/N_{B}} \end{array}$$

where $\delta_{M}$ and $\delta_{B}$ are the discretization errors corresponding to the $\phi_{M}$ and $\phi_{B}$, respectively. The on-grid algorithms, such as [6,7,8,9,10], can estimate the AoA/AoD effectively, by ignoring the discretization errors and assuming that the AoA/AoD are taken from a uniform grid of *N* points, i.e., $\phi \in \{-\frac{1}{2},-\frac{1}{2}+\frac{1}{N},\ldots ,\frac{1}{2}-\frac{1}{N}\},$ where *N* stands for the number of antennas and *ϕ* stands for the angle. In practice, the number of antennas is limited and the AoA/AoD are actually continuous, which causes the leakage of power, and, in turn, leads to the degradation of CE performance.

## 3. Off-Grid Channel Formulation

In order to suppress the negative effect caused by the off-grid problem, a specific and operable virtual channel representation model is introduced. From this virtual model, an enhanced off-grid angle estimation model is developed.

The continuously-distributed AoA can be written as:

(7) $$\begin{array}{c} \phi_{M}=-\frac{1}{2}+\frac{\phi_{M,k}}{N_{M}}+\frac{\delta_{M}}{N_{M}} \end{array}$$

where $\phi_{M,k}=k-1$ is the nearest *k*-th ($k=1,2,\ldots ,N_{M}$) discrete quantization grid of $\phi_{M}$, which is referred to as the integral grid angle, and $\left|\delta_{M}\right|<\frac{1}{2}$ stands for the deviation between $\phi_{M}$ and $\phi_{M,k}$, termed fractional off-grid angle. Similarly, the discrete form of the AoD is formulated as:

(8) $$\begin{array}{c} \phi_{B}=-\frac{1}{2}+\frac{\phi_{B,i}}{N_{B}}+\frac{\delta_{B}}{N_{B}} \end{array}$$

where $\phi_{B,i}=i-1$ ($i=1,2,\ldots ,N_{B}$) and $\left|\delta_{B}\right|<\frac{1}{2}$ are, respectively, the integral grid part and fractional off-grid part of the angle. Then, the fractional off-grid angle vectors $d_{M}\in C^{N_{M}\times 1}$ and $d_{B}\in C^{N_{B}\times 1}$ can be rewritten as:

(9) $$\begin{array}{c} d_{M}\left(\delta_{M}\right)={\left[1\;e^{j2\pi \delta_{M}/N_{M}}\cdots e^{j2\pi \left(N_{M}-1\right)\delta_{M}/N_{M}}\right]}^{T} \end{array}$$

(10) $$\begin{array}{c} d_{B}\left(\delta_{B}\right)={\left[1\;e^{j2\pi \delta_{B}/N_{B}}\cdots e^{j2\pi \left(N_{B}-1\right)\delta_{B}/N_{B}}\right]}^{T} \end{array}$$

Accordingly, the channel matrix in Equation (2) can be reformulated as:

(11) $$\begin{array}{cc} H & =\beta \text{Diag}{\left(d_{M}\left(\delta_{M}\right)\right)}^{T}a_{M}\left(-\frac{1}{2}+\frac{\phi_{M,k}}{N_{M}}\right)a_{B}^{T}\left(-\frac{1}{2}+\frac{\phi_{B,i}}{N_{B}}\right)\text{Diag}\left(d_{B}\left(\delta_{B}\right)\right)\\ & =D_{M}^{T}H_{D}D_{B} \end{array}$$

where $H_{D}=\beta a_{M}\left(-\frac{1}{2}+\frac{\phi_{M,k}}{N_{M}}\right)a_{B}^{T}\left(-\frac{1}{2}+\frac{\phi_{B,i}}{N_{B}}\right)$ represents the channel matrix only containing the integral grid part, and the diagonal matrices $D_{M}=\text{Diag}\left(d_{M}\left(\delta_{M}\right)\right)\in C^{N_{M}\times N_{M}}$ and $D_{B}=\text{Diag}\left(d_{B}\left(\delta_{B}\right)\right)\in C^{N_{B}\times N_{B}}$ represent the fractional off-grid angle matrixes. From the virtual channel model in the [18], $H_{D}$ can be rewritten as:

(12) $$\begin{array}{c} H_{D}=F_{M}^{T}{\mathrm{GF}}_{B} \end{array}$$

where $F_{B}\in C^{N_{B}\times N_{B}}$ is the discrete Fourier transform (DFT) matrix whose $\left(m_{B},n_{B}\right)$-th entry is $\frac{1}{\sqrt{N_{B}}}e^{j2\pi m_{B}\frac{n_{B}}{N_{B}}}$, $0\leq m_{B},\;n_{B}\leq N_{B}-1$, and $F_{M}\in C^{N_{M}\times N_{M}}$ is also a DFT matrix whose $\left(m_{M},n_{M}\right)$-th entry is $\frac{1}{\sqrt{N_{M}}}e^{j2\pi m_{M}\frac{n_{M}}{N_{M}}}$, $0\leq m_{M},\;n_{M}\leq N_{M}-1$. $G\in C^{N_{M}\times N_{B}}$ is referred to as the integral virtual channel matrix in angle domain with only one non-zero element, and ${\left[G\right]}_{k,i}$ denotes the nearest integral grid point to the actual angle. The row and column labels of the non-zero element, *k* and *i*, are connected to the $\phi_{M,k}$ and $\phi_{B,i}$ via

(13) $$\begin{array}{c} \phi_{M,k}=k-1 \end{array}$$

(14) $$\begin{array}{c} \phi_{B,i}=i-1 \end{array}$$

Then, the virtual representation model of the mmWave channel with continuously-distributed angle can be written as:

(15) $$\begin{array}{c} H=D_{M}^{T}F_{M}^{T}GF_{B}D_{B} \end{array}$$

where $D_{M}$ and $D_{B}$ are unitary matrices. It is interesting that the channel matrix H can be regarded as a two-dimensional (2D) orthogonal transform from the spatial domain to the beamforming domain. More specifically, the 2D orthogonal transform becomes the 2D DFT when the AoA/AoD locate at the quantization grids exactly.

## 4. The Off-Grid Turbo Channel Estimation Algorithm

In order to facilitate the operation of the proposed CE algorithm, the codebook designed in [17] is employed to divide the AoA/AoD estimation into two steps to estimate AoA and AoD separately at the BS and MS in adjacent time slots. Moreover, it can also be adopted here to estimate the fractional off-grid parts of AoA and AoD. Without loss of generality, the codebook design for AoA estimation at the MS is introduced as an example, and the same process can be employed at the BS to estimate the AoD. The process of training mode design is summarized as follows:

- **Training mode at the transmitter.** The training signal is designed to hold the form of $x=1_{K_{B}}$, and only one column of the beamforming matrix $W_{B}$ is chosen as ${\left[W_{B}\right]}_{:,n_{o}}=e_{N_{B}}\left(i_{o}\right)$, $\;n_{o}\in \left\{1,2,\cdots ,K_{B}\right\},\;i_{o}\in \left\{1,2,\cdots ,N_{B}\right\}$ while all other columns are zero vectors, where ${\left[W_{B}\right]}_{:,n_{o}}$ denotes the $n_{o}$-th column vector of matrix $W_{B}$. The training signals x are transmitted from $K_{B}$ RF chains and beamformed with the same $W_{B}$ in the successive *R* time slots.
- **Training mode at the receiver.** The combining matrix at the *r*-th time slot, $W_{M}^{r},r=1,2,\cdots ,R$, is designed in a way that its *n*-th column vector is ${\left[W_{M}^{r}\right]}_{:,n}=e_{N_{M}}\left(i_{n}^{r}\right),n=1,2,\cdots ,K_{M},$ where $\left\{i_{1}^{r},i_{2}^{r},\cdots ,i_{K_{M}}^{r}\right\}≜P_{M}^{r}\subset R_{M}^{r},$ with $P_{M}^{r}$ being randomly chosen from $R_{M}^{r}$ and $R_{M}^{1}≜\left\{1,2,\cdots ,N_{M}\right\}$, $R_{M}^{2}=\left\{1,2,\cdots ,N_{M}\right\}\setminus P_{M}^{1},\cdots ,R_{M}^{r}=\left\{1,2,\cdots ,N_{M}\right\}\setminus \left\{P_{M}^{1}\cup \cdots \cup P_{M}^{r-1}\right\}$.

In this way, the received signal at the MS can be written as:

(16) $$\begin{array}{c} y_{r}={\left(W_{M}^{r}\right)}^{T}D_{M}^{T}F_{M}^{T}{\mathrm{GF}}_{B}D_{B}W_{B}x+z \end{array}$$

Stacking $y_{r}$ into a vector, we have

(17) $$\begin{array}{cc} y & =[y_{1}^{T}\;y_{2}^{T}\;\cdots \;y_{R}^{T}]\\ & =WDFg+z \end{array}$$

where $W≜{\left[W_{M}^{1}\;W_{M}^{2}\cdots W_{MK}^{R}\right]}^{T}\in C^{RK\times N},$ with *N* and *K* being the number of antennas and RF chains, respectively. $F\in C^{N\times N}$ is a DFT matrix, and $g\in C^{N\times 1}$ is the integral virtual channel vector only containing the AoA of the LoS path seen by the MS. $D\in C^{N\times N}$ is the fractional off-grid angle matrix corresponding to the AoA. Apparently, W is a permutation matrix created by choosing *K* rows from an identity matrix I.

**Figure 2:**
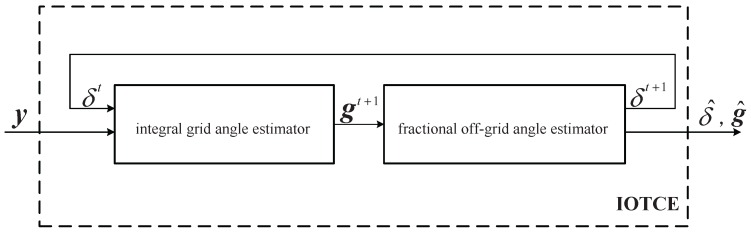
Flow chart of the iterative off-grid turbo channel estimation algorithm.

From Equation (17), the CE is accomplished by estimating the integral virtual channel, which, in turn, defines integral grid angle, and the fractional off-grid angle matrix, which stands for the fractional off-grid angle. As shown in Figure 2, an iterative off-grid turbo CE (IOTCE) algorithm is developed, which consists of two components: the integral grid angle estimator and the fractional off-grid angle estimator.

### 4.1. The Integral Grid Angle Estimator

Equation (17) degenerates into a CS recovery algorithm with W being the sampling matrix and D×F being the transform matrix when the fractional off-grid angle *δ* is given. As the application of the TCCS algorithm in [19], the ITCCS algorithm is adopted to iteratively estimate the integral virtual channel vector *g* from the Equation (17). $g\in C^{N\times 1}$ is a sparse signal to be estimated with *L* non-zero elements, following the Bernoulli–Gaussian distribution with $\lambda =\frac{L}{N}$ being the sparsity.

Let f≜Fg and q≜Df, and Equation (17) can be rewritten as:

(18) $$\begin{array}{c} y=Wq+z \end{array}$$

**Figure 3:**
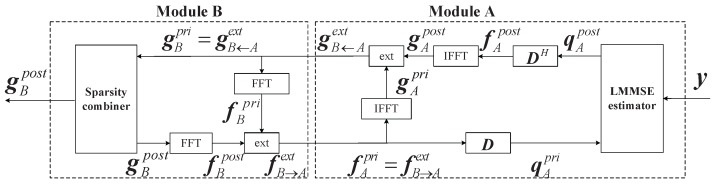
Flow chart of the improved turbo compressed channel sensing algorithm. FFT and IFFT denote the fast Fourier transform processing and the inverse transform processing, respectively. The modules D and $D^{H}$ denote the transform from f to q and the reverse processing.

As illustrated in Figure 3, the ITCCS algorithm consists of two modules: module A, the LMMSE estimator, and module B, the sparsity combiner. The LMMSE estimator provides an estimate of q by the linear minimum mean square estimation (LMMSE) method, from which an estimate of g can be obtained through constraints. Using the probability density estimation method, the sparsity combiner refines the estimate by exploiting the Bernoulli–Gaussian distribution. The specific realization of the algorithm can be seen in [19].

Therefore, by fixing *δ* and using the constraints Equations (9) and (10), we get the corresponding fractional off-grid angle vector d(δ) and matrix D, to estimate the integral virtual channel vector g at the sparsity $\lambda =\frac{L}{N}$ (L=1) using the ITCCS algorithm, where the value of *δ* is zero at the first iteration and is fed back from the fractional off-grid angle estimator at the iteration. For the purposes of this article, ITCCS $\left(W^{T},\text{Diag}\left(d^{t}\right),\frac{1}{N},\sigma^{2}\right)$ is used to make the description of the output, which is the estimate of g, and the parameters in brackets present the input of the algorithm.

### 4.2. The Fractional Off-Grid Angle Estimator

By fixing the integral virtual channel g, and as a result of the equivalence of an elementary row operation and premultiplication by a permutation matrix according to the theory of linear algebra, the Equation (17) can be rewritten as:

(19) $$\begin{array}{cc} y & =WDf+z\\ & ={\left[d\left(\delta \right)\right]}_{R}\circ {\left[f\right]}_{R}+z \end{array}$$

where $R=\{n_{1},n_{2},\ldots ,n_{K}\}$ is a set who is constituted by the row indices that are selected from I to W and the operator ∘ denotes the element-wise product. Equation (19) can be solved by the least square (LS) approach.

According to the proposed iteration, the IOTCE algorithm is illustrated in Algorithm 1, which presents a structure of a nested loop. In addition, the corresponding explanation of parameters in the IOTCE algorithm is listed in Table 1. The inner loop iterates the integral grid angle between sparsity and linearity, while the integral part and the fractional part are updated in turn during each iteration of the outer loop by following a fixed relationship. As the estimation of integral grid angle improves, the estimation of the fractional off-grid angle enhances via the outer loop.

**Algorithm 1** The Iterative Off-grid Turbo Channel Estimation Algorithm**Input:**
Received signal $y\in C^{RK\times 1}$;Sampling matrix $W\in C^{RK\times N}$, noise power $\sigma^{2}$.**Output:**
Estimate of g, $g^{t}$;Estimation of *δ*, $\delta^{t}$.**Iteration:**
**Step 1: Initialization**
$$\begin{array}{c} t=0,\delta^{0}=0 \end{array}$$
**Step 2: Estimation of *g***
(1) Fixing *δ*,
$$\begin{array}{cc} d^{t} & ={\left[1\;e^{j2\pi \delta^{t}/N}\;e^{j2\pi 2\delta^{t}/N}\cdots e^{j2\pi \left(N-1\right)\delta^{t}/N}\right]}^{T}\\ g^{t+1} & =\text{ITCCS}(W^{T},\text{Diag}\left(d^{t}\right),\frac{1}{N},\sigma^{2}) \end{array}$$

**Step 3: Estimation of** *δ*
(1) Fixing g,
$$\begin{array}{c} {\left[d^{t+1}\right]}_{R}=y⊘{\left[g^{t+1}\right]}_{R} \end{array}$$
where ⊘ denotes the element-wise division operation.(2) Calculating $\delta^{t+1}$ by linear fitting after phase extraction from the equation,
$$\begin{array}{c} {\left[d^{t+1}\right]}_{R}={\left[e^{j2\pi \left(n_{1}-1\right)\delta^{t+1}/N}\;e^{j2\pi \left(n_{2}-1\right)\delta^{t+1}/N}\cdots e^{j2\pi \left(n_{K}-1\right)\delta^{t+1}/N}\right]}^{T} \end{array}$$

**Step 4: Iteration of loop**
**IF** iteration stopping condition is satisfied
output the estimated value.
**ELSE**
t=t+1.Then, and turn to Step 2.



## 5. Simulation Results

In this section, we evaluate the performance of the proposed algorithm in the mmWave communication systems. The simulation system consists of a BS and an MS, and the same ULA with $N_{B}=N_{M}=N$ half-wavelength spaced antennas and $K_{B}=K_{M}=K$ RF chains is configured at both the BS and MS. Typical mmWave channels featuring much stronger LoS paths than NLoS paths are simulated, and a one-path channel model is used to approximate the typical mmWave channel in the low signal-to-noise ratio (SNR) region. The fading weight of the path follows Rayleigh distribution with variance 1, and the continuously-valued AoA/AoD are uniformly-distributed in the range $\left[-\frac{\pi }{2},\frac{\pi }{2}\right)$.

**Figure 4:**
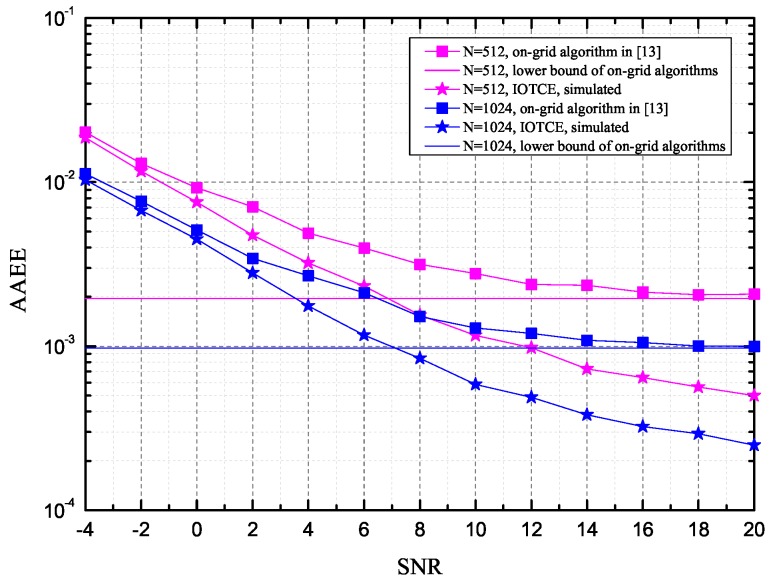
The average angle estimation error (AAEE) versus signal-to-noise ratio (SNR) for the proposed algorithm for different values of *N*.

The simulation results of the average angle estimation error (AAEE), defined as $E[|\phi -\hat{\phi }|],$ with $\hat{\phi }$ being the estimate of the true angle *ϕ*, are presented in Figure 4, where the on-grid algorithm in [17] and the lower bound of the AAEE for on-grid algorithms, which is $\frac{1}{N}$, are also presented for reference in two cases of N=1024 and N=512. From the curves in Figure 4, we can observe that the proposed method outperforms the on-grid method and achieves AAEE of 10^−2^ at SNR *γ* = −4 dB. As expected, the AAEE of the proposed algorithm exceeds the lower bound of the on-grid algorithms as SNR increases, which validates the AAEE performance of our algorithm. For example, the gaps between the simulated AAEE and the lower bound are 0.00139 and 0.0007 when *γ* = 20 dB for N=512 and N=1024, respectively. The gap shrinks from 0.00139 to 0.0007 by the increase in *N*, because the less sparsity ($\lambda =\frac{1}{N}$) can achieve a the better recovery performance at the given samplings according to the theory of CS [20].

**Figure 5:**
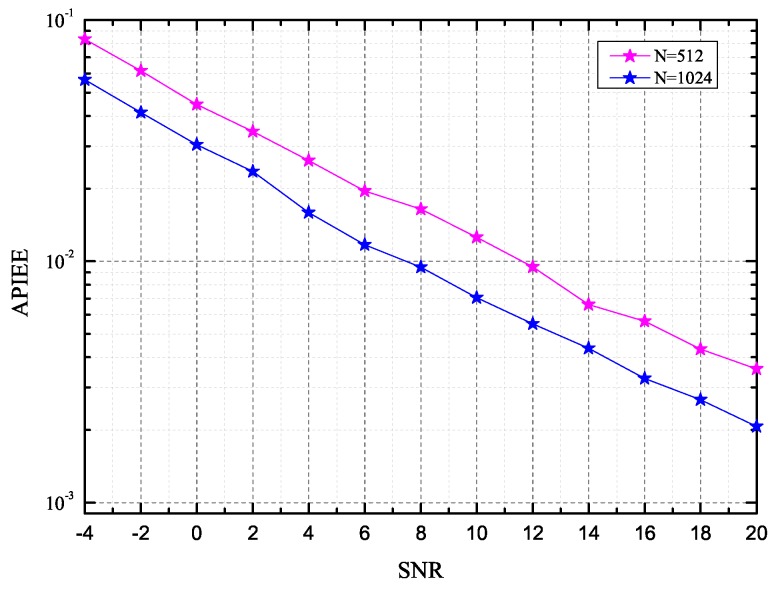
The average probability of integral grid point estimation error (APIEE) versus signal-to-noise ratio (SNR) for the proposed algorithm for different values of *N*.

The average probability of integral grid point estimation error (APIEE) performance, which is defined as $E[I\left(\phi_{k}\neq {\hat{\phi }}_{k}\right)],$ with $\hat{\phi_{k}}$ being the estimate of the real integral grid angle $\phi_{k}$, is also simulated, and the simulation results are illustrated in Figure 5. As clearly shown, the bigger *N* leads to the better APIEE performance at a given *K*. For example, at the APIEE of 10^−2^, the algorithm with N=1024 almost enhances by 4 dB with respect to N=512.

## 6. Conclusions

The shortage of spectrum at present will be alleviated by advances in the mmWave communication systems, and directional precoding/beamforming with large antenna arrays appears to be inevitable to support longer outdoor links and to provide sufficient received signal power than before. In this paper, an enhanced CE algorithm is developed for the off-grid problem in the mmWave systems with massive antenna arrays and RF chain constraints. First, we developed an off-grid formulation to catch the quantization problem. Then, an iterative method, which depends on the developed off-grid formulation is proposed to renew and upgrade the CE between the integral and fraction angle information under the Turbo principle. Simulation results show the efficiency and high resolution of the proposed method. For future work, it would be more practical to consider the multi-paths channel with independently but not identically distributed paths, and develop an efficient method to calculate the Log Likelihood Ratio (LLR) information.

## Figures and Tables

**Table 1:** Explanation of parameters in the iterative off-grid turbo channel estimation algorithm.

Parameters	Explanation
*N*	the number of antennas at receiver
g	the integral virtual channel vector
W	the combining matrix at receiver
*δ*	the fractional off-grid angle
d	the corresponding fractional off-grid angle
